# Surgery

**Published:** 1878-12

**Authors:** 


### SURGERY.

### FOREIGN BODY IN THE RECTUM: SUCCESSFUL REMOVAL. *Hospitals Tidende.* (*Canad. Jour. Med. Sci.*)

A man, æt. 35, introduced into the rectum, open end uppermost, a preserve bottle nearly seven inches long, for the purpose of stopping a diarrhœa. The next morning he complained of pain; chloroform was given, and the bottle, which could before this be felt in the rectum, passed higher up. It could now be felt through the abdominal walls, in the middle line, with the bottom just above the pubis. He was again anæsthetized and posterior linear rectotomy performed; but efforts to reach the bottle in this way were unavailing. Abdominal section was then made in the linea alba. The gut was divided over the bottle, and removal slowly effected. The neighboring parts were protected from escape of fæces by sponges and compresses. The gut was closed with catgut sutures. Recovery was slow and complicated by local peritonitis and abscesses; but the patient was discharged *cured* in less than fourteen weeks.

The reporter, Dr. Studsgaard, refers to three other cases of a similar character reported by Ogle, Closmadeuc and Reali; this, with the others, making a group of rare and curious cases.

Action of Parenchymatous Injections of Glacial Acetic Acid on Carcinoma. Dr. Gies. (*Centralblatt f. Chir., No.* 19.)

The author injected diluted glacial, acetic acid (1 : 3 aq. destil.) into a recurrent carcinoma, as large as a hen's egg, seated in the right side of the inferior maxilla; the injection excited suppuration, and the tumor was diminished to the size of a hazelnut. A primary carcinoma, as large as a hen's egg, situated beneath the ear of the same patient, was treated in the same way, and after 21 days had almost entirely disappeared; 25 syringefuls were injected before this tumor suppurated. A carcinoma as large as a hen's egg, situated in the left breast of a woman, suppurated after 10 injections, and in the course of a month had shrunken to a nodule about the size of a hazlenut.

Treatment of Burns.

Many of the journals of the day have allusions to the use of a solution of sodic bicarbonate as a local application to burned surfaces, however large. Some of their readers have tried solutions of varying strength, and not a few have written that they were disappointed. In order to produce the desired effect, it is necessary to use a *saturated* solution, 1 pt. to 6, or thereabouts, and apply it on lint or other suitable dressing. This in many cases gives almost instantaneous relief.

